# Effects of Wheat Bug (*Eurygaster* spp. and *Aelia* spp.) Infestation in Preharvest Period on Wheat Technological Quality and Gluten Composition

**DOI:** 10.1155/2014/148025

**Published:** 2014-01-15

**Authors:** Aleksandra M. Torbica, Jasna S. Mastilović, Milica M. Pojić, Žarko S. Kevrešan

**Affiliations:** Institute of Food Technology, University of Novi Sad, Bulevar Cara Lazara 1, 21000 Novi Sad, Serbia

## Abstract

The effects of wheat bug infestation (*Eurygaster* spp. and *Aelia* spp.) on the composition of wheat gluten proteins and its influence on flour technological quality were investigated in the present study. Wheat samples of six wheat varieties, collected from two localities in northern Serbia, were characterized by significantly different level of wheat bug infestation. Composition of wheat gluten proteins was determined using sodium dodecyl sulphate polyacrylamide gel electrophoresis (SDS PAGE), while the selected parameters of technological quality were determined according to standard and modified empirical rheological methods (Farinograph, Extensograph, Alveograph, and Gluten Index). The surface morphology of the selected samples was viewed using scanning electron microscopy (SEM). Wheat from wheat bug-infested locality regardless of the variety had deteriorated technological quality expressed with higher Farinograph softening degree, lower or immeasurable Extensograph energy, and Alveograph deformation energy. The most important changes in the gluten proteins composition of bug-infested wheat were related to gliadin subunits with molecular weights below 75 kDa, which consequently caused deterioration of uniaxial and biaxial extensibility and dough softening during mixing.

## 1. Introduction

Wheat bugs (*Eurygaster* spp. and *Aelia* spp.) have been the regular members of entomofauna of small grains in Serbia. In this regard, in years characterized by shorter and milder winter, sudden transitions from low to high daily temperatures, and/or occasional local occurrence of heat stresses in May and June, the critical number of insects is exceeded [1]. The same phenomenon has been observed in several semiarid regions around the world—South and Eastern Europe, North Africa, Middle East, and New Zealand [2, 3]. Wheat bug infestation alters the gluten status of wheat kernel, where wheat bug-proteinase affects the disruption of gluten complex thus influencing the deterioration of rheological properties of wheat dough, poor baking performance, and ultimately unsatisfactory final product appearance [4, 5]. The degree of such deterioration depends on the intensity of wheat bug infestation and is often accompanied by significant economic damage [5–9]. Therefore, the substantial efforts have been made so far by wheat breeders both to explain the resistance mechanism and methods to determine resistance to wheat bugs and to select the potential gene sources to utilize the resistance to wheat bug infestation in wheat breeding. In this way, an important contribution to agricultural economy has been made equally beneficial to wheat producers, millers, and bakers [10–12].

Gluten is a specific protein fraction of wheat responsible for viscoelastic properties of wheat dough. It is considered as a functional ingredient that influences breadmaking performance and the quality of final products [13]. Gliadin (Gli) and glutenin (Glu) fractions of wheat gluten comprise functional part of the total wheat proteins with divergent role in determining the rheological properties of dough [14–16]. Since glutenins impart elasticity and gliadins impart viscosity and extensibility to dough, the overall rheological properties are dependent on their quantity, composition, and ratio. Gluten quality is predominantly influenced by genotype and also by crop agronomy and the presence of biotic and abiotic factors [9, 17–19]. Rheological properties of dough of bug-damaged wheat are characterized by lower Farinograph dough development time, dough stability, mixing tolerance index [6, 20], and higher softening degree [20]. Moreover, the decrease in Alveograph deformation energy, tenacity, and extensibility [6, 20] as well as Extensograph energy [21] has been registered. Bug-damaged wheat is also characterized by lower Gluten Index (GI) as determined by standard and/or modified method [21, 22].

The aim of this study was to analyse the influence of wheat bug damage on selected technological properties of wheat varieties and to relate them with gluten complex composition.

## 2. Materials and Methods

### 2.1. Materials

Six winter wheat varieties: Cipovka (1), Kantata (2), Jefimija (3), Dragana (4), Sofija (5), and Pobeda (6), were collected from two geographically close localities in northern Serbia (A: 19°46′ East and 46°06′ North at an altitude of 102 m and B: 20°28′ East and 45°51′ North at an altitude of 81 m). The appearance of wheat bug infestation was registered in Locality A, while the application of insecticides protected wheat from wheat bug infestation in Locality B.

### 2.2. Methods

The content of bug-damaged kernels was determined according to ICC standard method number 102/1 [23]. Wheat samples were milled using a Bühler MLU 202 laboratory mill with flour extraction rate of 60%. The rheological properties of wheat dough were determined by Farinograph, Extensograph, Alveograph, and Gluten Index according to ICC standard methods [23]. Modified Gluten Index, extraction of glutenins and gliadins from flour samples, and sodium dodecyl sulphate polyacrylamide electrophoresis (SDS-PAGE) were performed as previously described by Torbica et al. [21]. The number of bands and relative amount of glutenin and gliadin subunits were observed within the following ranges of molecular weights: below 40 kDa, 40–80 kDa, and above 80 kDa for glutenins and below 30 kDa, 30–75 kDa, and above 75 kDa for gliadins and expressed as a percentage of total area within each electropherogram [24–27].

The surface morphology of the flour samples was viewed using a scanning electron microscopy (SEM), where sample preparation included coating with gold using a sputter coating device (Baltec SCD 005). Micrographs were obtained using a SEM-Jeol JSM 6460LV instrument with a magnification of 1000x.

### 2.3. Statistical Analysis

Statistical data analysis was performed using Statistica software, version 10.0. The significance of differences between the selected technological quality indicators and gluten composition was tested by one-way ANOVA.

## 3. Results

**Figure 1:**
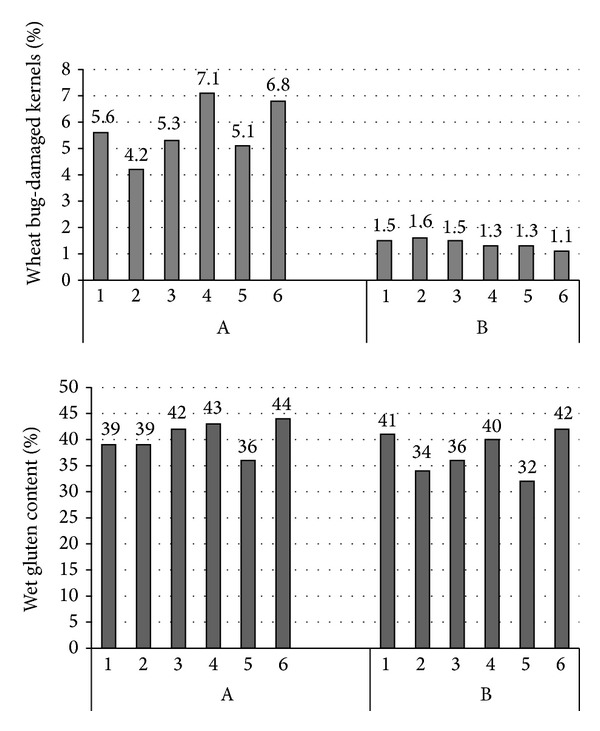
Comparison of observed varieties (1–6) and localities (A and B) with respect to wheat bug infestation and wet gluten content.

The differences in the growing conditions and applied crop agronomy in the two localities resulted in significant differences in the content of wheat bug damage kernels which was from three (Variety 2) to over five (Variety 6) times higher in Locality A compared to Locality B. However, significant differences between localities were not registered in terms of wet gluten content ranging from 30 to over 40% (Figure 1). Differences in the amount of wheat bug-damaged kernels (WBK) yielded the significant differences in the selected rheological properties of wheat dough (Table 1). Specifically, the poor protein functionality of samples harvested in Locality A was expressed by higher Farinograph softening degree (SD), lower Extensograph energy (*E*), and lower Alveograph deformation energy (*W*) in comparison to those of the samples from the Locality B. However, the quality of wheat grown in the Locality A was so much deteriorated that Alveograph and Extensograph tests for the majority of examined varieties could not be performed due to the poor dough properties (assigned value 0). Moreover, significantly lower standard (GIS) and modified (GIM) Gluten Index values (*P* ≤ 0.05) were observed for all varieties from the Locality A indicating low protein functionality that could be attributed to wheat bug infestation [18] (Table 1). Statistically significant differences in the technological quality of wheat varieties from observed localities were noted for all examined wheat varieties, with the exception of Variety 6 in the case of SD (Table 1).

**Figure 2:**
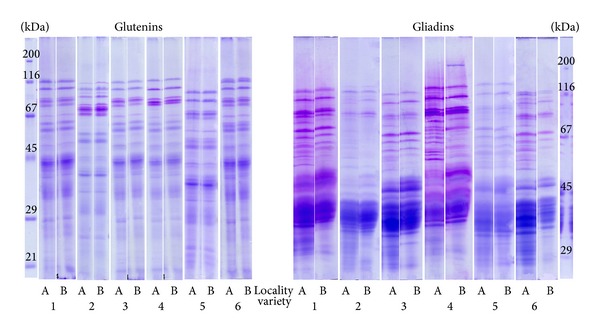
Glutenins and gliadins electropherograms of examined wheat varieties (1–6) from observed localities (A and B).

SDS PAGE electropherograms of glutenins and gliadins generally show the larger number of gliadin bands of wheat varieties grown in the Locality A (Figure 2). Nevertheless, the noticeable differences in the number of glutenin bands were not registered. Data obtained by SDS PAGE including the number of bands and the amount of glutenin and gliadin subunits within the predefined range of molecular masses are shown in Tables 2 and 3.

Significant differences in the total amount of glutenins (Glu) and gliadins (Gli) between localities were not registered regardless of the variety (*P* > 0.05) (Tables 2 and 3). Nevertheless, gliadin to glutenin ratio (Gli/Glu) ranged from 0.67 to 1.13 and from 0.60 to 0.96 for wheat varieties grown in Locality A and Locality B, respectively (data not shown). All varieties grown in Locality A exhibited higher Gli/Glu than those from Locality B, with the exception of Variety 6. All varieties grown in the Locality A showed significant increase in the amount and number of bands of gliadin with molecular weights under 30 kDa. Oppositely, wheat bug infestation caused the decrease in the amount and number of bands of gliadins with molecular weights in the range 30–75 kDa. The number of gliadin bands above 75 kDa generally increased with the exception of Variety 1, which exhibited the lowest infestation rate. The number of glutenin bands for the majority of varieties within all the molecular weight ranges showed no particular trend indicating that the number of glutenin bands was influenced by the variety itself (Table 3). The difference in the relative amount of glutenin subunits with molecular weights below 40 kDa was not observed between the localities, whereas Varieties 5 and 6 exhibited higher relative amount of those glutenin subunits in Locality A due to the highest infestation rate (Figure 1, Table 2). The variety with the lowest WBK (Variety 1) showed the difference between the localities in the relative amount of glutenins in molecular weight range 40–80 kDa. Statistically significant decrease in the relative amount of glutenins with molecular weight above 80 kDa was registered only for Varieties 1 and 6.

Scanning electron micrographs of endosperm particles obtained from selected wheat variety from both localities (Figure 2) illustrate the change in microstructure of the endosperm of the bug-damaged kernels.

## 4. Discussion

Although a significant difference in wet gluten content between the localities was not observed, the change in the composition of gluten complex was confirmed by significantly different values of Gluten Index obtained by both standard and modified method (Table 1) [21]. These findings were in accordance with findings of Aja et al. [28] who indicated that wet gluten content of bug-damaged wheat remains constant, whereas the Gluten Index of damaged gluten showed a steady decrease with the different incubation times implying gluten protein hydrolysis. The quality and functionality of gluten proteins are associated with the presence or absence of specific gluten protein fractions-glutenins and gliadins, their total amount, and their ratio, where each fraction has a specific role in the formation of viscoelastic properties of wheat dough [29]. The gliadin proteins contribute to the viscosity and extensibility of dough, whereas glutenins are responsible for dough strength and elasticity [30, 31]. The protein complex degradation due to the proteolytic process caused by wheat bug attack resulted in higher number of gliadin bands of molecular weights below 30 kDa and in range 30–75 kDa (Figure 2, Table 3). That was followed by the increase in relative amount of gliadins with molecular weight below 30 kDa and decrease in relative amount of gliadins in range of molecular weight 30–75 kDa (Table 3). Accordingly, it seems that the most important changes in the gluten proteins composition in bug-infested wheat were related to gliadin subunits with molecular weights below 75 kDa, which presumably altered the viscoelastic properties of dough. The rheological properties of wheat dough are influenced by the ratio of wheat gluten fractions, by the physicochemical bonds between them and their interactions [32]. In the case when the changes in the amount of gliadin subunits were accompanied with the increasing amount of total gliadins, these changes also resulted in extreme increase in Farinograph SD (Varieties 1–5). Obtained results indicated that more intensive wheat bug infestation rate might have been related to gradual decomposition of glutenins resulting in higher relative amount of glutenins of low molecular weights [33].

**Figure 3:**
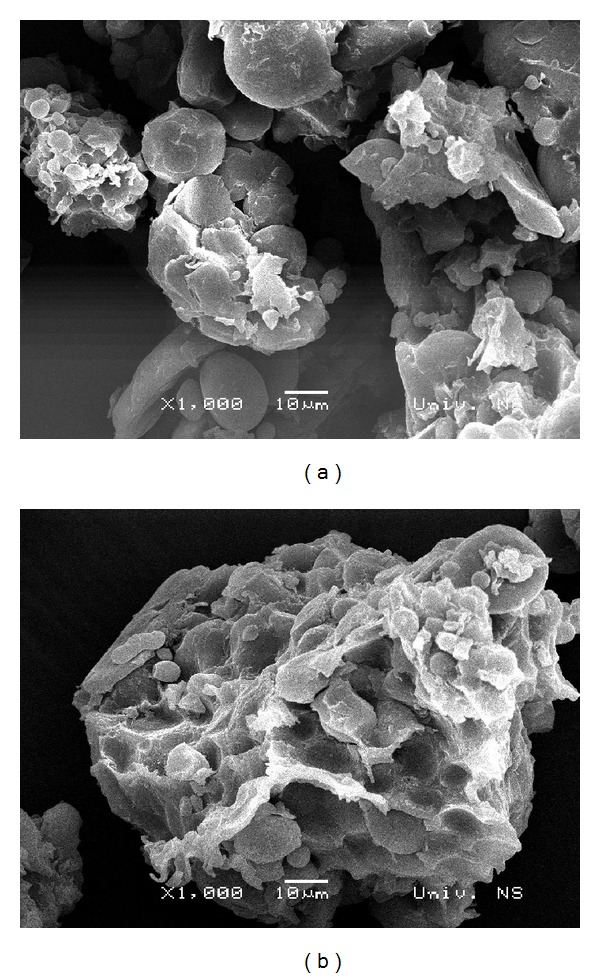
Comparison of micrographs (1000x) of bug-damaged kernel endosperm (a) and the same variety (2) without bug infestation (b).

The microstructure of kernel endosperm (Figure 3) reflected the quality and the shape of protein matrix. Scanning electron micrograph of endosperm particle of selected wheat variety (Variety 6) grown in Locality B (Figure 3(b)) showed more compact protein matrix structure with the starch granules closely embedded in. In contrast, the endosperm of bug-damaged kernel grown in Locality A (Figure 3(a)) was characterized by poorly cohesive structure due to damaged protein matrix [3, 8].

Wheat bug infestation affected decrease in Gluten Index values significantly determined by both standard and modified Gluten Index methods indicating the change in the composition of gluten complex. The proteolytic process degraded gluten towards creation of higher number of both glutenin and gliadin bands, but the most expressed change was related to gliadins of molecular weights below 75 kDa. The change in the number of bands and amount of gliadin and glutenin fractions affected the technological quality of selected wheat varieties, altering uniaxial and biaxial extensional properties as well as dough mixing properties.

## Figures and Tables

**Table 1:** Comparison of technological quality of wheat varieties between selected localities.

Wheat variety	Locality	GIS	GIM	SD (BU)	*E* (cm^2^)	*W* (J10^−4^)
1	A	66,67^a^	9,87^a^	175^a^	10^a^	0^a^
	B	82,35^b^	48,40^b^	100^b^	85^b^	194^b^
2	A	47,22^a^	0,00^a^	175^a^	0^a^	0^a^
	B	71,88^b^	41,18^b^	70^b^	32^b^	218^b^
3	A	52,38^a^	0,00^a^	215^a^	0^a^	0^a^
	B	86,11^b^	40,49^b^	40^b^	55^b^	189^b^
4	A	48,72^a^	0,29^a^	190^a^	0^a^	0^a^
	B	75,61^b^	34,83^b^	95^b^	30^b^	147^b^
5	A	34,09^a^	0,00^a^	195^a^	0^a^	0^a^
	B	66,67^b^	31,05^b^	40^b^	33^b^	228^b^
6	A	39,53^a^	0,00^a^	90^a^	0^a^	0^a^
	B	80,00^b^	49,52^b^	90^a^	55^b^	186^b^

GIS: Gluten Index standard, GIM: Gluten Index modified, SD: Farinograph softening degree, *E*: Extensograph energy, and *W*: Alveograph deformation energy.

Values followed by different letters in the same column are significantly different from each other (*P* < 0.05).

**Table 2:** The number of bands and relative amount of glutenin subunits within the selected molecular weight ranges for wheat varieties from observed localities.

Wheat variety	Locality	Glu (%)	<40 kDa		40–80 kDa		>80 kDa	
			*n*	(%)	*n*	(%)	*n*	(%)
1	A	54,25^a^	12	32.1^a^	20	43.1^a^	10	24.4^a^
	B	55,89^a^	16	39.9^a^	15	27.1^b^	9	33.2^b^
2	A	47,01^a^	14	35.7^a^	15	44.8^a^	6	19.7^a^
	B	51,33^a^	11	39.8^a^	12	45.0^a^	6	15.1^a^
3	A	50,64^a^	15	34.1^a^	16	45.7^a^	6	20.2^a^
	B	52,47^a^	13	36.2^a^	11	46.9^a^	6	16.8^a^
4	A	47,20^a^	18	30.5^a^	15	44.4^a^	8	25.2^a^
	B	50,93^a^	13	30.8^a^	13	44.2^a^	7	24.2^a^
5	A	54,16^a^	18	46.0^a^	18	37.4^a^	9	17.0^a^
	B	62,56^a^	16	36.5^b^	13	42.5^a^	8	20.9^a^
6	A	60,05^a^	8	37.3^a^	18	47.2^a^	10	15.3^a^
	B	57,26^a^	10	23.9^b^	21	51.9^a^	6	24.1^b^

Values followed by different letters in the same column are significantly different from each other (*P* ≤ 0.05).

**Table 3:** The number of bands and relative amount of gliadin fractions within the selected molecular weight ranges for wheat varieties from observed localities.

Wheat variety	Locality	Gli (%)	<30 kDa		30–75 kDa		>75 kDa	
			*n*	(%)	*n*	(%)	*n*	(%)
1	A	45,75^a^	9	26.4^a^	28	66.5^a^	9	6.7^a^
	B	44,11^a^	5	8.6^b^	26	79.3^b^	12	12.2^a^
2	A	52,99^a^	6	11.8^a^	24	82.6^a^	12	5.4^a^
	B	48,67^a^	5	2.8^b^	19	91.1^b^	8	6.2^a^
3	A	49,36^a^	8	11.5^a^	22	73.5^a^	10	14.8^a^
	B	47,53^a^	4	0.0^b^	21	86.4^b^	9	13.6^a^
4	A	52,80^a^	7	1.1^a^	29	61.1^a^	13	37.9^a^
	B	49,07^a^	6	0.0^b^	21	76.9^b^	12	23.2^b^
5	A	45,84^a^	7	18.2^a^	22	72.3^a^	13	9.4^a^
	B	37,44^a^	7	4.7^b^	19	84.5^b^	7	10.7^a^
6	A	39,95^a^	4	18.7^a^	23	78.1^a^	12	3.3^a^
	B	42,74^a^	3	5.9^b^	21	89.3^b^	8	4.9^a^

Values followed by different letters in the same column are significantly different from each other (*P* ≤ 0.05).

## References

[1] Stamenković S (2004 (Serbian)). Visoka brojnost žitnih stenica na mestima prezimljavanja u jesen 2003. godine. *Biljni Lekar*.

[2] Sivri D, Köksel H, Bushuk W (1998). Effects of wheat bug (*Eurygaster maura*) proteolytic enzymes on electrophoretic properties of gluten proteins. *New Zealand Journal of Crop and Horticultural Science*.

[3] Pérez G, Bonet A, Rosell CM (2005). Relationship between gluten degradation by *Aelia* spp and *Eurygaster* spp and protein structure. *Journal of the Science of Food and Agriculture*.

[4] Kara M, Sivri D, Köksel H (2005). Effects of high protease-activity flours and commercial proteases on cookie quality. *Food Research International*.

[5] Hariri G, Williams PC, El-Haramein FJ (2000). Influence of pentatomid insects on the physical dough properties and two-layered flat bread baking quality of syrian wheat. *Journal of Cereal Science*.

[6] Karababa E, Ozan AN (1998). Effect of wheat bug (*Eurygaster integriceps*) damage on quality of a wheat variety grown in Turkey. *Journal of the Science of Food and Agriculture*.

[7] Köksel H, Atli A, Dag A, Sivri D (2002). Commercial milling of suni bug (*Eurygaster* spp.) damaged wheat. *Nahrung*.

[8] Rosell CM, Aja S, Sadowska J (2002). Amylase activities in insect (*Aelia* and *Eurygaster*)-damaged wheat. *Journal of the Science of Food and Agriculture*.

[9] Hristov N, Mladenov N, Djuric V, Kondic-Spika A, Marjanovic-Jeromela A, Simic D (2010). Genotype by environment interactions in wheat quality breeding programs in southeast Europe. *Euphytica*.

[10] Kinaci E, Kinaci G (2007). Genotypic variations in yield and quality of wheat damaged by Sunn pest (*Eurygaster* spp.). *Pakistan Journal of Botany*.

[11] El Bouhssini M, Street K, Joubi A, Ibrahim Z, Rihawi F (2009). Sources of wheat resistance to Sunn pest, *Eurygaster integriceps* Puton, in Syria. *Genetic Resources and Crop Evolution*.

[12] Mehrabadi M, Bandani AR, Saadati F, Ravan S (2009). Sunn pest, *Eurygaster integriceps* Putton (Hemiptera: Scutelleridae), digestive *α*-amylase, *α*-glucosidase and *β*-glucosidase. *Journal of Asia-Pacific Entomology*.

[13] Ćurić D, Karlović D, Tušak D, Petrović B, Đugum J (2001). Gluten as a standard of wheat flour quality. *Food Technology and Biotechnology*.

[14] Southan M, MacRitchie F (1999). Molecular weight distribution of wheat proteins. *Cereal Chemistry*.

[15] Kuktaite R, Larsson H, Johansson E (2004). Variation in protein composition of wheat flour and its relationship to dough mixing behaviour. *Journal of Cereal Science*.

[16] Jakubauskiene L, Juodeikiene G (2005). The relationship between protein fractions of wheat gluten and the quality of ring-shaped rolls evaluated by the echolocation method. *Food Technology and Biotechnology*.

[17] Triboï E, Abad A, Michelena A, Lloveras J, Ollier JL, Daniel C (2000). Environmental effects on the quality of two wheat genotypes: 1. Quantitative and qualitative variation of storage proteins. *European Journal of Agronomy*.

[18] Torbica A, Živančev D, Hadnađev M, Mastilović J Influence of heat stress on wheat grain quality.

[19] Torbica A, Živančev D, Mastilović J, Knežević D, Bodroža-Solarov M Impact of changes in climate conditions on the technological quality of wheat.

[20] Zoccatelli G, Vincenzi S, Corbellini M, Vaccino P, Tavella L, Curioni A, Lafiandra D, Masci S, D'Ovidio R (2004). Breakdown of glutenin polymers during dough mixing by *Eurygaster maura* protease. *The Gluten Proteins*.

[21] Torbica A, Antov M, Mastilović J, Knežević D (2007). The influence of changes in gluten complex structure on technological quality of wheat (*Triticum aestivum* L.). *Food Research International*.

[22] Torbica A, Mastilović J (2008). Influence of different factors on wheat proteins quality. *Food Processing, Quality and Safety*.

[23] (1996). *ICC Standard Methods of the International Association for Cereal Science and Technology *.

[24] Lindsay MP, Skerritt JH (1999). The glutenin macropolymer of wheat flour doughs: structure-function perspectives. *Trends in Food Science and Technology*.

[25] Gianibelli MC, Larroque OR, MacRitchie F, Wrigley CW (2001). Biochemical, genetic, and molecular characterization of wheat glutenin and its component subunits. *Cereal Chemistry*.

[26] Shewry PR, Halford NG (2002). Cereal seed storage proteins: structures, properties and role in grain utilization. *Journal of Experimental Botany*.

[27] Sebastiano R, Simó-Alfonso EF, Citterio A, Ramis-Ramos G (2004). Prediction of wheat dough W and P/L inflation test parameters by capillary zone electrophoresis of a protein extract followed by multivariate regression. *Electrophoresis*.

[28] Aja S, Pérez G, Rosell CM (2004). Wheat damage by *Aelia* spp. and *Erygaster* spp.: effects on gluten and water-soluble compounds released by gluten hydrolysis. *Journal of Cereal Science*.

[29] Wieser H, Kieffer R (2001). Correlations of the amount of gluten protein types to the technological properties of wheat flours determined on a micro-scale. *Journal of Cereal Science*.

[30] Wang YG, Khan K, Hareland G, Nygard G (2006). Quantitative glutenin composition from gel electrophoresis of flour mill streams and relationship to breadmaking quality. *Cereal Chemistry*.

[31] Wieser H (2007). Chemistry of gluten proteins. *Food Microbiology*.

[32] Lásztity R (1975). Rheological properties of wheat gluten and their relation to molecular parameters. *Die Nahrung*.

[33] Salis L, Goula M, Valero J, Gordún E (2010). Prolamin proteins alteration in durum wheat by species of the genus *Eurygaster* and *Aelia* (Insecta, Hemiptera). *Spanish Journal of Agricultural Research*.

